# Celastrol Suppresses Glioma Vasculogenic Mimicry Formation and Angiogenesis by Blocking the PI3K/Akt/mTOR Signaling Pathway

**DOI:** 10.3389/fphar.2020.00025

**Published:** 2020-02-06

**Authors:** Yingjun Zhu, Xihong Liu, Peiyuan Zhao, Hui Zhao, Wei Gao, Lei Wang

**Affiliations:** ^1^ School of Traditional Chinese Medicine, Beijing Key Lab of TCM Collateral Disease Theory Research, Capital Medical University, Beijing, China; ^2^ Basic Discipline of Integrated Chinese and Western Medicine, Henan University of Chinese Medicine, Henan, China; ^3^ School of Pharmaceutical Sciences, Capital Medical University, Beijing, China; ^4^ Advanced Innovation Center for Human Brain Protection, Capital Medical University, Beijing, China

**Keywords:** celastrol, glioblastoma, vasculogenic mimicry, angiogenesis, VE-cadherin

## Abstract

Angiogenesis and vasculogenic mimicry (VM) are thought to be the predominant processes ensuring tumor blood supply during the growth and metastasis of glioblastoma (GBM). Celastrol has potential anti-glioma effects, however the mechanisms underlying these effects remain unclarified. Recent studies have shown that the PI3K/Akt/mTOR signaling pathway is closely related to angiogenesis and VM formation. In the present study, we have demonstrated, for the first time, that celastrol eliminated VM formation by blocking this signaling pathway in glioma cells. By the treatment of celastrol, tumor growth was suppressed, tight junction and basal lamina structures in tumor microvasculature were disarranged in U87 glioma orthotopic xenografts in nude mice. Periodic acid Schiff (PAS)-CD31 staining revealed that celastrol inhibited both VM and angiogenesis in tumor tissues. Additionally, celastrol reduced the expression levels of the angiogenesis-related proteins CD31, vascular endothelial growth factor receptor (VEGFR) 2, angiopoietin (Ang) 2 and VEGFA, VM-related proteins ephrin type-A receptor (EphA) 2, and vascular endothelial (VE)-cadherin. Hypoxia inducible factor (HIF)-1α, phosphorylated PI3K, Akt, and mTOR were also downregulated by treatment with celastrol. *In vitro*, we further demonstrated that celastrol inhibited the growth, migration, and invasion of U87 and U251 cells, disrupted VM formation, and blocked the activity of PI3K, Akt, and mTOR. Collectively, our data suggest that celastrol inhibits VM formation and angiogenesis likely by regulating the PI3K/Akt/mTOR signaling pathway.

## Introduction

Glioblastoma (GBM), which is characterized by rapid growth and high aggressiveness, is the most aggressive type of brain tumor (Louis et al., 2016). With the incidence rate of 2.1 per million for all populations 20–59 years old and the overall survival time for GBM is 14.4 months in China (Yang et al., 2013; Thakkar et al., 2014; Jiang et al., 2016). The comprehensive strategy for the treatment of GBM involves surgical resection and chemo-radiotherapy with temozolomide (TMZ), but some infiltrative tumor cells often reside in areas away from the primary tumor, and cannot be eliminated by operation (Chauffert et al., 2014). These residual glioma cells can form vasculogenic mimicry (VM) channels, leading to glioma recurrence. In the last few years, VM has been regarded as a mechanism producing a specific blood supply for the growth and metastasis of tumors (Wang et al., 2013). VM channels have been detected in high-grade gliomas and are substantially associated with the degree of tumor malignancy, in addition, patients with VM-positive gliomas have shorter life expectancies than those with VM-negative gliomas. VM structures can express endothelial cell (EC)-related genes and mimic ECs, providing a convenient conduit for glioma perfusion and invasion (Liu et al., 2011).

In GBM, angiogenesis is generally thought to be the predominant process for tumor growth and metastasis, and it is mediated primarily through the vascular endothelial growth factor receptor (VEGFR) pathway (Arnone et al., 2018). However, many small-molecule VEGFR inhibitors, such as sorafenib and cediranib, have failed to provide any survival benefits to GBM patients (Agarwal et al., 2011). VM has been identified to participate in anti-angiogenesis therapy resistance in GBM (Chen and Cleck, 2009; Xu et al., 2012; Soda et al., 2013; Simon et al., 2018). VM, which is characterized by the formation of vascular channels lined by glioma cells, is believed to be a new blood passage method different from angiogenesis (Yue and Chen, 2005). There are no ECs in the lumens of VM channels, but basement membrane-like structures form, that are positive for periodic acid-Schiff (PAS) staining and negative for platelet EC adhesion molecule-31 (PECAM-1/CD31) staining (Chen and Chen, 2014). When angiogenesis cannot fulfill the blood supply needs of a tumor, tumor cells may utilize their plasticity to form VM channels and exhibit EC phenotypes under hypoxic conditions. Therefore, endovascular drugs with anti-VM effects need to be used to improve therapeutic benefits for patients with GBM.

Celastrol is a triterpenoid derived from *Tripterygium wilfordii* Hook F, a Chinese herbal medicine used to treat idiopathic refractory nephrotic syndrome, rheumatoid arthritis, Crohn's disease, and moderate to severe psoriasis vulgaris (Xu et al., 2009; Marks, 2011; Wu et al., 2015; Zhu et al., 2015; Zhou et al., 2019). Recently, experimental evidence has shown that celastrol inhibits the growth of xenografts of various type of cancers, including desmoplastic melanoma, prostate cancer, and ovarian cancer (Yang et al., 2006; Liu et al., 2018; Xu et al., 2019). Additionally, celastrol abolishes NF-κB activation in human triple-negative breast cancer (TNBC) and HepG2 cells, induces apoptosis of pancreatic cancer cells, oral cancer cells, and A549 cells (Shrivastava et al., 2015; Shen et al., 2016; Ding et al., 2017; Lin et al., 2019; Zhang et al., 2019). In addition, it promotes the autophagic degradation of EGFR in non-small cell lung cancer (NSCLC), inhibits growth and angiogenesis in prostate tumors by suppressing the protein kinase B (Akt)/mammalian target of rapamycin (mTOR)/P70S6K pathway (Pang et al., 2010; Xu et al., 2016). Celastrol also suppresses the growth of subcutaneous glioma xenografts and reduces angiogenesis by interrupting the expression of VEGFRs (Huang et al., 2008). Furthermore, celastrol inhibits vascular EC proliferation, migration, and tube formation *in vitro* and decreases micro-vessel density (MVD) in a SHG-44 subcutaneous model (Zhou and Huang, 2009). However, the effects of celastrol on VM formation and their mechanisms have not been reported. Our study examined, for the first time, whether celastrol can eliminate VM formation in glioma and explored the underlying mechanism.

Mutation of the phosphoinositide 3-kinase (PI3K)/Akt/mTOR signaling pathway is related to cell proliferation, metabolism, apoptosis, and angiogenesis in GBM (Thorne et al., 2016; Binder et al., 2018). Our previous studies have indicated that celastrol inhibits C6, U87, and U251 cell growth and induces apoptosis partly by blocking the Akt/mTOR signaling pathway (Liu et al., 2019). Some research has also demonstrated that inhibition of the PI3K/Akt/mTOR signaling pathway can disrupt VM channels in SHG-44 and U251 cells (Choi et al., 2014; Zhang et al., 2015). Through extensive literature review, we found that Ephrin type-A receptor (EphA) 2 and vascular endothelial (VE)-cadherin are essential proteins required for VM formation (Paulis et al., 2010). VE-cadherin regulates EphA2 activity, and EphA2 modulates the p85 regulatory subunit of PI3K, promoting the loss of tumor intercellular adhesion and facilitating cell migration and infiltration to form VM channels (Kim et al., 2019; Brantley-Sieders et al., 2004).

Based on the above findings, we propose that celastrol may disrupt glioma VM channels through the PI3K/Akt/mTOR signaling pathway. In our present study, the inhibitory effects of celastrol on VM formation, angiogenesis, and the related PI3K/Akt/mTOR signaling pathway were investigated in a model of U87 glioma orthotopic xenografts and in U87 and U251 cells. TMZ, which is widely used as a non-specific DNA alkylating agent in glioma treatment, was used as a positive control for anti-tumor effects in our study.

## Materials and Methods

### Establishment and Treatment of an Intracranial Glioma Model in Nude Mice

This research was approved by the Animal Experiment and Experimental Animal Welfare Committee of Capital Medical University (AEEI-2016-097). Male BALB/c-nu mice (18–20 g, 8 weeks old) were purchased from Beijing Vital River Laboratory Animal Technology Co., Ltd. (SCXK [Jing] 2016-0011) and housed in specific pathogen-free (SPF) conditions with constant temperature (22 ± 3°C) and humidity (40–50%) at the Experimental Animal Center of Capital Medical University. The animals were provided with standard solid rodent chow and water. A volume of 5 μl of serum-free medium containing 5 × 10^5^ U87 cells (Cell Resource Center, IBMS, CAMS/PUMC, Beijing, China) was injected into the right striatum of each treated nude mouse using a stereotaxic apparatus (RWD Life Science, Shenzhen, China). Sham-operated mice were manipulated in the same way but injected with serum-free medium without U87 cells. After 4 days, the mice were divided randomly into six groups based on a random number list (a random number sequence was generated by: www.randomizer.com): the sham-operated (Sham) group, the model (MO) group, the TMZ (Sigma, MO, USA)-treated (TMZ) group, and three celastrol (0.5, 1, and 2 mg/kg, Pharmacodia Co., Ltd., Beijing, China)-treated groups (Lin et al., 2015). There were eight mice in each group. The mice in the Sham and MO groups were injected intraperitoneally with phosphate-buffered saline (PBS) containing 1% DMSO (0.1 ml/10 g). The mice in the TMZ group were injected with TMZ at 20 mg/kg (Pandey et al., 2019), and the mice in the celastrol-treated groups were injected with celastrol dissolved in PBS containing 1% DMSO every other day (Yang et al., 2017). The weight of each mouse was recorded during treatment (**Figure S1**).

### Magnetic Resonance Imaging (MRI) Examination

T2-weighted MRI (T2WI) was performed on the brains of the mice in each group (n = 4) on the 19th day after implantation. Images of 25 tumor coronal brain sections (slice thickness: 0.3 mm) were obtained by rapid acquisition with a relaxation enhancement (Turbo-RARE) sequence (TE/TR = 45/4,000 ms) for observation and to choose appropriate slices for measurement (Lajous et al., 2018). The tumor volume was recorded as the sum of the tumor infiltration area multiplied by the thickness of each slice, as calculated with ImageJ software (Li et al., 2018).

### Histopathology Staining

After the MRI experiment, mice from each group (n = 3) were anesthetized with 4% chloral hydrate, perfused with 4% paraformaldehyde, and embedded with paraffin and 5 μm thick sections were prepared. A section was chosen at the coronal plane of tumor inoculation site. The tumor histopathology of the brain was assessed with hematoxylin and eosin (H&E) staining. Additionally, H&E staining of the heart, liver, and spleen was used to evaluate celastrol toxicity (**Figure S2**).

### Transmission Electron Microscopy (TEM)

For TEM observation, a mouse from each group (n = 1) was perfused with 4% paraformaldehyde containing 2% glutaraldehyde for 40 min. Then, normal brain tissues from Sham mouse tissues and tumor tissues were cut into 1 mm^3^ pieces and fixed in 2.5% glutaraldehyde for 2 h. After fixation with 2% osmium, the samples were dehydrated and embedded, and ultrathin sections were visualized under a JEM-1400 transmission electron microscope.

### PAS-CD31 Dual Staining

For immunohistochemical staining of CD31, slices were dewaxed, hydrated, and then subjected to tissue antigen recovery in citric acid buffer for 20 min at 95°C. After blocking with 2% BSA (9048-46-8, VWR Life Science, CA, USA), the sections were incubated with mouse anti-CD31 (ab24590, 1:200) at 4°C overnight. A horseradish peroxidase (HRP)-labeled secondary antibody (rabbit anti-mouse IgG) was added and the sections were incubated at 37°C for 1 h. Then, the slices were washed with PBS. After staining with 3,3′-diaminobenzidine (DAB), the slices were washed with distilled water, stained with periodic acid for 5 min, washed with PBS, incubated with Schiff reagent for an additional 15 min, and finally counterstained with hematoxylin. Endothelium-dependent vessels (EVs) were evaluated by counting the CD31-positive vessels (Wang et al., 2018). The average numbers of PAS-stained vessels and EVs at the peripheral zone of tumor necrosis were calculated in 10 randomly selected fields.

### Bioinformatics Analysis

The correlation between the expression of the VM-related protein EphA2 in GBM tissues and the survival rate was analyzed with the Gene Expression Profiling Interactive Analysis (GEPIA) database (Tang et al., 2017).

### Cell Culture

Two human glioma cell lines U87 and U251 (Cell Resource Center, IBMS, CAMS/PUMC, Beijing, China) were used in this study. U87 cells were cultured in MEM-NEAA (Corning, NY, USA) supplemented with 10% fetal bovine serum (FBS, HyClone, Logan, UT, USA) and a 1% penicillin-streptomycin solution. U251 cells were cultured in the medium mentioned above without NEAA. Astrocytes were derived from primary cortex cultures from newborn rats (Liu et al., 2019). The cells were maintained with 5% CO_2_ in a humidified incubator at 37°C.

### Cell Counting Kit (CCK)-8 Assay

U87, U251 cells, and astrocytes (1.0 ×10^4^) were seeded in 96 well plates for 24 h and the cells were treated with celastrol (0.125, 0.25, 0.5, 1, and 2 μM) for 24 and 48 h. Then, cell viability was analyzed by CCK-8 assay according to the provided protocol. The optical density (OD) values were detected by a microplate reader at 450 nm and normalized to those of the control groups.

### Wound Healing Assay

Glioma cells were synchronized by serum withdrawal for 24 h prior to the wound healing assay to minimize gap closure bias. The cells were administrated with celastrol (0.25, 0.5, and 1 μM) when fully confluent. Then, a line was scratched into each cell monolayer with a sterile 200 μl pipette tip, and the same field was photographed immediately afterwards and 24 h later. The area of the gap at 0 h (*S_0_*) and 24 h (*S_24_*) was measured with ImageJ software, and the closure was subsequently determined as *S_0_-S_24_*.

### Cell Migration Assay

A Transwell migration assay was performed using 2 × 10^4^ glioma cells in serum-free medium. After cells were seeded into Transwell chamber in 24-well plates, the lower chambers were filled with 500 μl of MEM with 10% FBS as a chemoattractant. After incubation with celastrol (0.25, 0.5, and 1 μM) for 24 h, the cells were fixed with 4% paraformaldehyde for 30 min and then dyed with 0.5% crystal violet solution for 10 min. After washing with PBS, the cells on top of the Transwell chamber were wiped off. The migrating cells were calculated in five randomly selected fields.

### Cell Invasion Assay

A Matrigel basement membrane matrix (Corning, NY, USA) was diluted with MEM to 2 mg/ml and then laid on Transwell chamber. After rehydration at 37°C for 1 h, the chambers were seeded with glioma cells. The remaining steps of the Transwell invasion assay were carried out as in the Transwell migration assay.

### VM *In Vitro*

To observe the effects of celastrol on VM *in vitro*, U87 and U251 cells (2×10^5^ cells/ml, 100 μl) were cultured in Matrigel-coated 24 well plates with celastrol (0, 0.25, 0.5, and 1 μM) or LY294002 (CST, 9901) and SC79 (MCE, HY-18749). The tumor cells were incubated at 37°C for 24 h. Images of VM tubule structures were obtained by phase-contrast microscopy. The average numbers of master junctions and total segment lengths in five different fields were analyzed using ImageJ software (Choi et al., 2014).

### PAS Staining

After VM formation occurred *in vitro*, glioma cells were fixed in 100% ethanol for 1 h and incubated with 0.5% PAS. After counterstaining with hematoxylin, images of PAS staining were captured by phase-contrast microscopy.

### Immunofluorescence (IF) Analysis

Slices were dewaxed, hydrated, and then subjected to tissue antigen recovery in citric acid buffer for 20 min. After blocking with 2% BSA, the slices were incubated with the following primary antibodies overnight at 4°C: goat anti-zonula occluden (ZO)-1 (ab190085, 1:100), rabbit anti-caveolin-1 (ab18199, 1:200), mouse anti-CD31 (1:100), rabbit anti-VE-cadherin (ab33168, 1:200), and rabbit anti-EphA2 (6997T, 1:200). Then, the slices were rewarmed at 37°C for 1 h and washed with PBS. Subsequently, the slices were incubated with the corresponding secondary antibodies (Alexa Fluor 488 donkey anti-rabbit IgG [1:500]/Cy3-labeled donkey anti-goat IgG [1:500], Alexa Fluor 488 goat anti-rabbit IgG [1:400], Alexa Fluor 594 goat anti-mouse IgG [1:400]) for 2 h and washed with PBS. Finally, the slices were mounted with Fluoro-Gel with DIPI. Fluorescence images of the slices were obtained with laser confocal scanning microscope and a Leica fluorescence microscope (Leng et al., 2018). The expression of the above proteins was analyzed in five fields randomly selected fields at the peripheral zone of tumor necrosis by ImageJ software.

Cells (6×10^4^) were seeded in 20 mm confocal dishes, cultured for 24 h, and then treated with celastrol (0, 0.25, 0.5, and 1 μM) or LY294002 (CST, 9901) and SC79 (MCE, HY-18749) for 24 h. These cells were fixed with 4% paraformaldehyde for 30 min and treated with 0.5% Triton for 10 min. After washing with filtered PBS, the cells were blocked with 5% BSA for 1 h and incubated with anti-VE-cadherin antibody at 4°C overnight. Then, the cells were washed with PBST (PBS with 0.05% Tween 20) and incubated with Alexa Fluor 488 donkey anti-rabbit IgG secondary antibody (1:500) for 1 h while protected from light. Finally, the cells were dyed with DAPI for 5 min and washed with PBST again three times before being photographed under laser confocal scanning microscopy. The number of positive stained cells was normalized to the total number of cells, which was set as 100%. All the images were analyzed with ImageJ software by an investigator who was blinded to the experimental groups.

### Western Blot (WB) Analysis

Cells (treated with drugs according to the same method for IF analysis) and tissues from the marginal areas of tumors (n = 3 per group) were lysed with RIPA lysis buffer. The supernatant was collected after centrifugation, and the protein concentrations were normalized using a Bicinchoninic Acid (BCA) Protein Assay Kit. The proteins were separated by SDS-PAGE and transferred onto PVDF membranes. After blocking with Tris-base buffered saline with 0.1% Tween-20 (TBST) containing 5% skim milk for 1 h, the membranes were incubated with the following primary antibodies overnight at 4°C: mouse anti-actin (66009, 1:1,000), anti-ZO-1 (1:800), anti-caveolin-1 (1:1,000), rabbit anti-VEGFR2 (ab11939, 1:1,000), rabbit anti-angiopoietin (Ang)2 (ab155106, 1:2,000), rabbit anti-VEGFA (YT5108, 1:2,000), anti-EphA2 (1:1,000), anti-VE-cadherin (1:800), mouse anti-hypoxia inducible factor (HIF)-1α (ab113642, 1:1,000), rabbit anti-PI3K (4257T, 1:1,000), rabbit anti-phospho-PI3K (4228T, 1:1,000), rabbit anti-Akt (4691T, 1:1,000), rabbit anti-phospho-Akt (4060T, 1:1,000), rabbit anti-mTOR (ab32028, 1:1,000), and rabbit anti-phospho-mTOR (ab109268, 1:1,000). The membranes were washed with TBST four times for 5 min and then incubated with HRP-linked secondary antibodies (1:10,000). The proteins were exposed using enhanced chemiluminescence (ECL) reagent (Zhao et al., 2018). The greyscale values of the bands were detected by ImageJ software.

### Statistical Analysis

Statistical analyses were performed with one-way analysis of variance (ANOVA) and Tukey's HSD multiple comparisons tests using GraphPad Prism 7.0 software. The data are presented as the mean ± the standard error of the mean (SEM) of at least three independent experiments (n ≥ 3). *P*-values <0.05 were considered to indicate statistical significance.

## Results

### Celastrol Suppressed the Growth and Infiltration of Glioma Orthotopic Xenografts in Nude Mice

**Figure 1:**
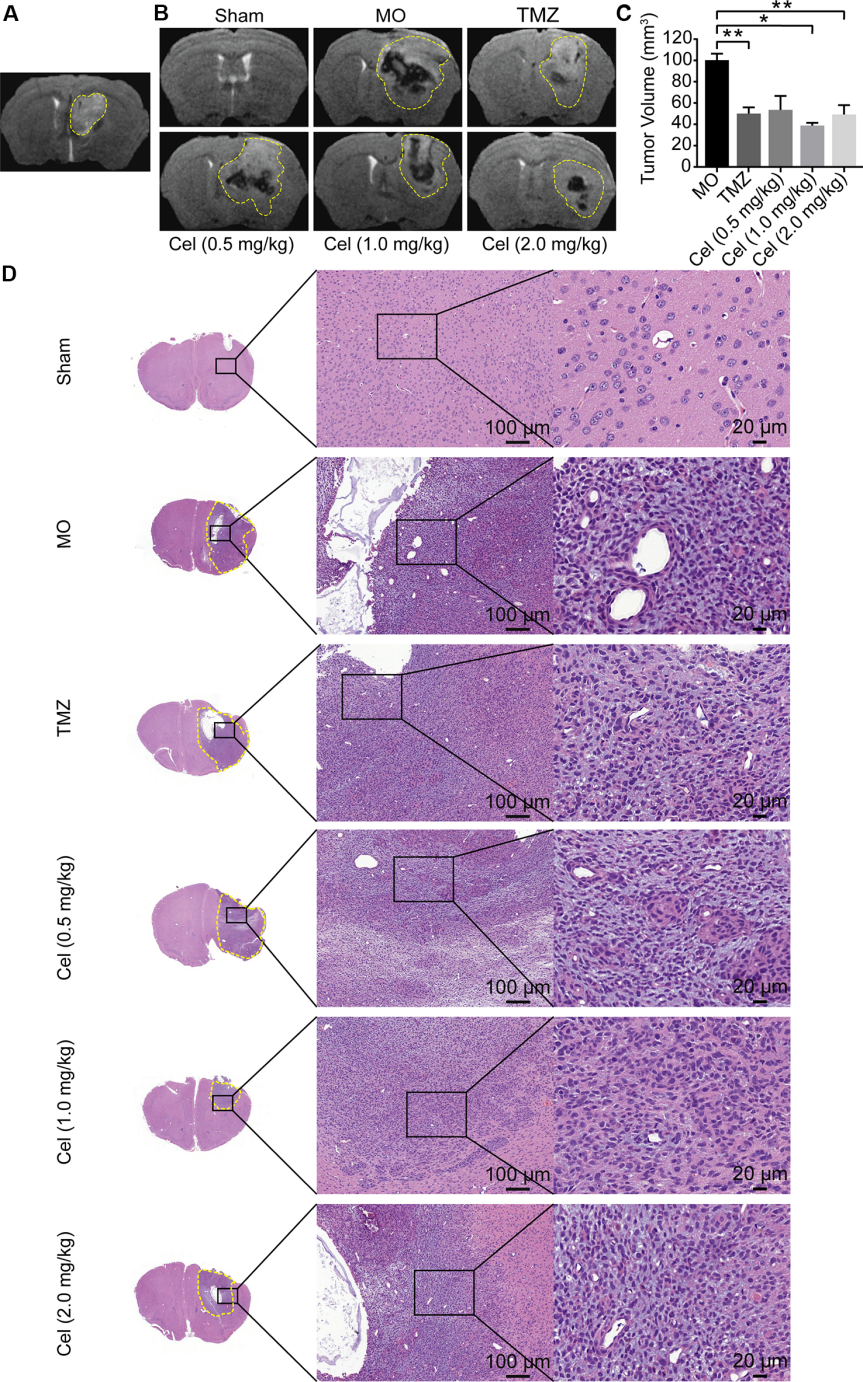
Celastrol (Cel) suppressed the tumor growth and infiltration of orthotopic U87 glioma xenografts in nude mice. **(A)** Four days after inoculation, T2-weighted Magnetic Resonance Imaging (T2WI) of coronal brain sections showed the formation of tumor in the model mice. **(B, C)** On the 19th day after implantation, T2WI of coronal brain sections, temozolomide (TMZ) and Cel (1 and 2 mg/kg) inhibited tumor growth as analyzed by tumor volume. **(D)** Hematoxylin and eosin staining of solid tumors revealed that TMZ- and Cel-treated mice had inhibited tumor infiltration. The data are expressed as the mean ± SEM of three independent experiments, *P < 0.05, **P < 0.01 *vs* model (MO) group.

U87 orthotopic xenograft models were used to probe the inhibitory effects of celastrol on glioma *in vivo*. As described in our previous study, tumor formation in the caudate nucleus was observed on the fifth day, indicating that the model was successfully established (**Figure 1A**), so celastrol treatment was started at this point. Analysis of coronal T2WI images indicated that tumor volumes were substantially lower in the mice receiving celastrol doses of 1 and 2 mg/kg than in the MO mice (**Figures 1B, C**) and that the inhibitory effects of celastrol were similar to those of TMZ. H&E staining of mouse brains revealed the histopathology of glioma xenograft tissues. Necrosis, abnormal microvascular proliferation, high cell densities, cellular atypia, and mitotic figures were observed in MO mice. TMZ- and celastrol-treated mice had reduced tumor cell density and invasiveness (**Figure 1D**). No significant difference in body weight was observed between celastrol-treated mice and MO mice (**Figure S1**). H&E staining of mouse hearts, livers, and kidneys suggested that celastrol did not cause major organ-related toxicity (**Figure S2**). Hence, the MRI and H&E staining analyses suggested that celastrol suppressed the progression of glioma in mice at doses of 1 and 2 mg/kg.

### Celastrol Disrupted the Microvascular Structure of Glioma Orthotopic Xenografts

**Figure 2:**
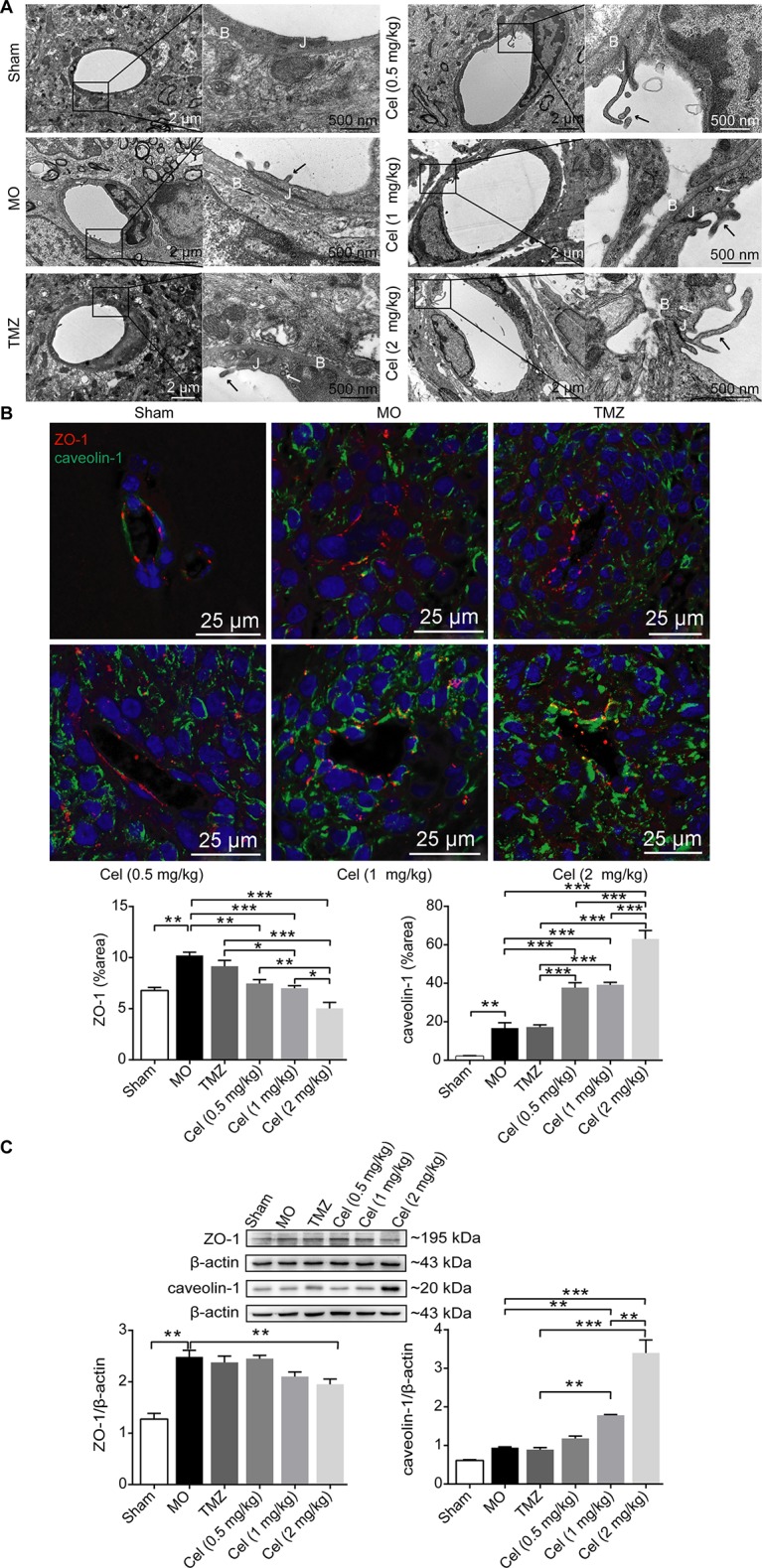
Celastrol (Cel) disrupted the microvascular structure of glioma orthotopic xenografts. **(A)** Microvascular morphology in the tumors imaged by Transmission Electron Microscopy revealed that Cel disrupted the tight junctions (J) and the basal lamina structure (B) of microvasculature, vesicles (white arrows) and membrane protrusions (black arrows) linked to endothelial cells in the tumor microvascular lumen were also observed. **(B)** Immunofluorescence localization of ZO-1 and caveolin-1 in tumor microvasculature. ZO-1 (red) and caveolin-1 (green) were labelled with fluorescent secondary antibodies, and the nuclei were labelled with DAPI. **(C)** Western blot analysis of ZO-1 and caveolin-1 expression. The data are expressed as the mean ± SEM of three independent experiments, *P < 0.05, **P < 0.01, ***P < 0.001.

Failure of drug penetration through the blood brain barrier (BBB) is one of the main causes of poor responses of glioma patients to clinical systemic therapies (Walter et al., 2015), so we investigated the effect of celastrol on the microvascular structures in the BBB. TEM revealed the histopathology of U87 glioma xenograft tissues at the ultrastructural level. The microvasculature in the BBB was formed by ECs with tight junctions in Sham mice, and the vessels were surrounded by a basal lamina and astrocytic end feet. Tight junctions were still intact in MO mice, but the basal laminas in MO mice were more irregular and thicker than those in Sham mice. Several membrane protrusions linked to ECs in tumor capillary lumens were observed in MO mice. The basal laminas of capillaries and the contacts between the astrocyte feet and capillaries were disrupted in mice treated with celastrol at doses of 1 and 2 mg/kg, and more intracellular vesicles and membrane protrusions were observed in these capillary lumens than in those of MO mice (**Figure 2A**). To test the effects of celastrol on tumor microvascular structures, IF staining of the tight junction-related protein ZO-1 and the endocytosis-related protein caveolin-1 was performed. ZO-1 exhibited a continuous distribution at the edges of ECs, while caveolin-1 was expressed in the cytoplasm in tumor tissues. The expression of ZO-1 and caveolin-1 was higher in MO mice than in Sham mice (**Figure 2B**). Compared with MO and TMZ mice, celastrol-treated mice had a discontinuous distribution and low expression of ZO-1, while caveolin-1 expression was significantly higher in celastrol-treated mice than in MO and TMZ mice. Then, ZO-1 and caveolin-1 expression levels were determined by WB analysis. ZO-1 expression was substantially higher in MO mice than in Sham mice (**Figure 2C**) but was significantly lower in 2 mg/kg celastrol-treated mice than in MO mice. Caveolin-1 expression was substantially higher in 1 and 2 mg/kg celastrol-treated mice than in MO and TMZ mice, in addition, compared with 1 mg/kg celastrol, 2 mg/kg celastrol substantially increased caveolin-1 expression. Hence, our data suggest that celastrol promote its own transport to tumor tissues by disrupting microvascular structures and promoting the caveolin-1-related transcellular pathway.

### Celastrol Inhibited VM Formation and Angiogenesis of Glioma Orthotopic Xenografts

**Figure 3:**
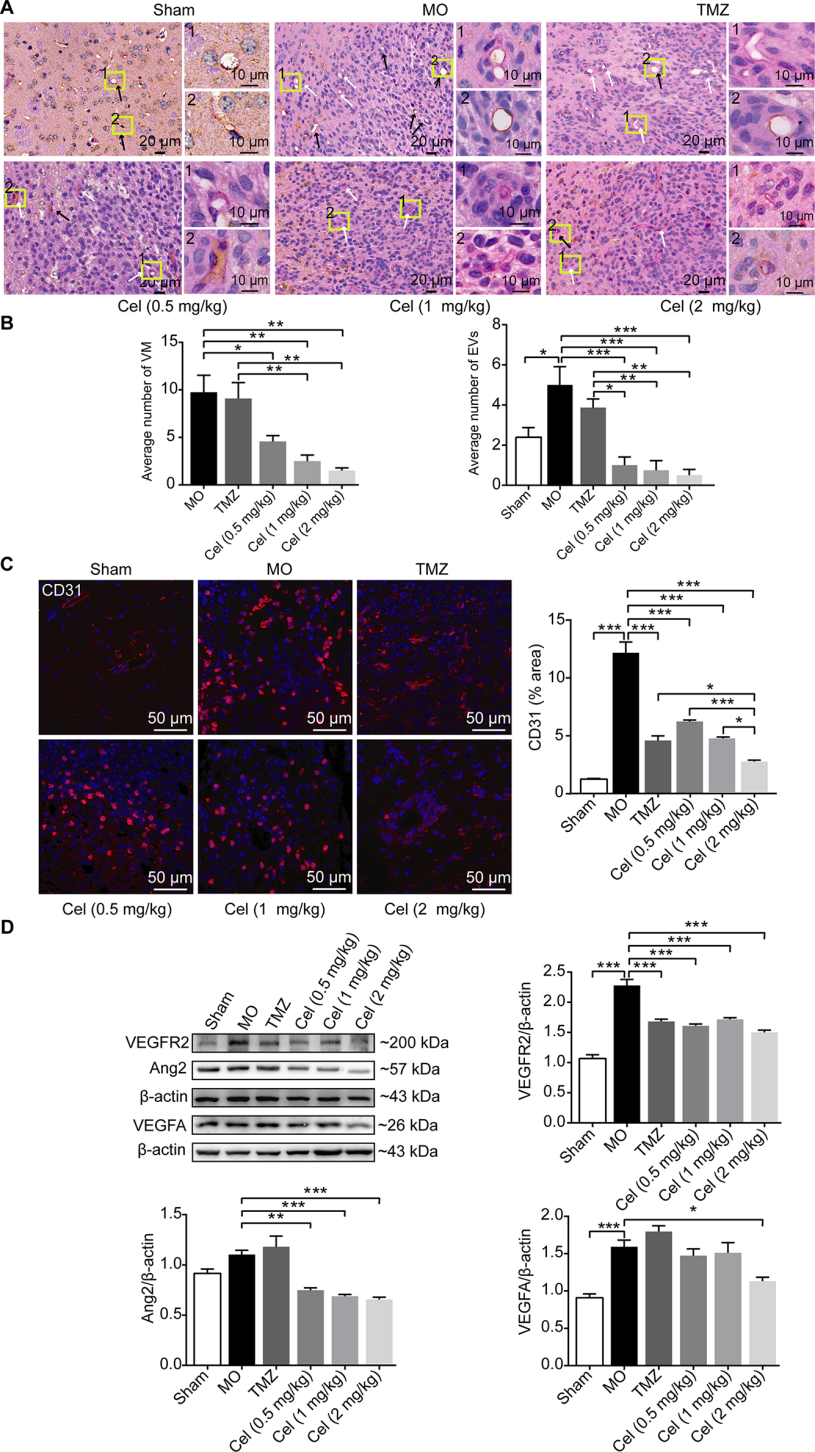
Celastrol inhibited VM formation and angiogenesis of glioma orthotopic xenografts. **(A, B)** PAS-CD31 dual staining of tumor microvasculature. The white arrows indicate periodic acid-Schiff-positive staining, and the black arrows indicate EVs. **(C)** Immunofluorescence localization of CD31 in tumor vessels. CD31 (red) was labelled with fluorescent secondary antibodies, and the nuclei were labelled with DAPI. **(D)** Western blot analysis of VEGFR2, Ang2, VEGFA expressions in tumor tissues. The data are expressed as the mean ± SEM of three independent experiments, *P < 0.05, **P < 0.01, ***P < 0.001.

To observe the effect of celastrol on glioma VM formation *in vivo*, PAS-CD31 dual staining was performed to distinguish VM channels and EVs. In MO mice, VM was characterized as being present when PAS staining was positive and CD31staining was negative. EVs were confirmed by CD31 positivity (Ricci-Vitiani et al., 2010). VM channels were lined by tumor cells containing red blood cells in vascular lumens, indicating their functional connection with EVs (Soda et al., 2011). There were obviously fewer VM channels in celastrol-treated mice than in MO and TMZ mice (**Figures 3A, B**). The higher number of EVs in MO mice than in Sham mice indicated microvascular proliferation in glioma tissue. There were significantly fewer EVs in celastrol-treated mice than in MO and TMZ mice, and IF of CD31 proved this effect (**Figure 3C**). Then, the anti-angiogenic effects of celastrol were detected by WB analysis of VEGFR2, Ang2, and VEGFA expression. VEGFR2 and VEGFA were both present at substantially higher levels in MO mice than in Sham mice (**Figure 3D**). While, no obvious difference of Ang2 expression was detected between these two group, we speculated VEGFA may play a dominant role in angiogenesis. VEGFR2 and Ang2 expression was notably lower in celastrol-treated mice than in MO mice. Celastrol at a dose of 2 mg/kg effectively reduced the expression of VEGFA. Overall, VM and angiogenesis coexisted in glioma orthotopic xenografts, and both methods of vascularization in tumors were inhibited by celastrol.

### Celastrol Decreased the Expression of the VM-Related Proteins EphA2 and VE-cadherin in Glioma Orthotopic Xenografts

**Figure 4:**
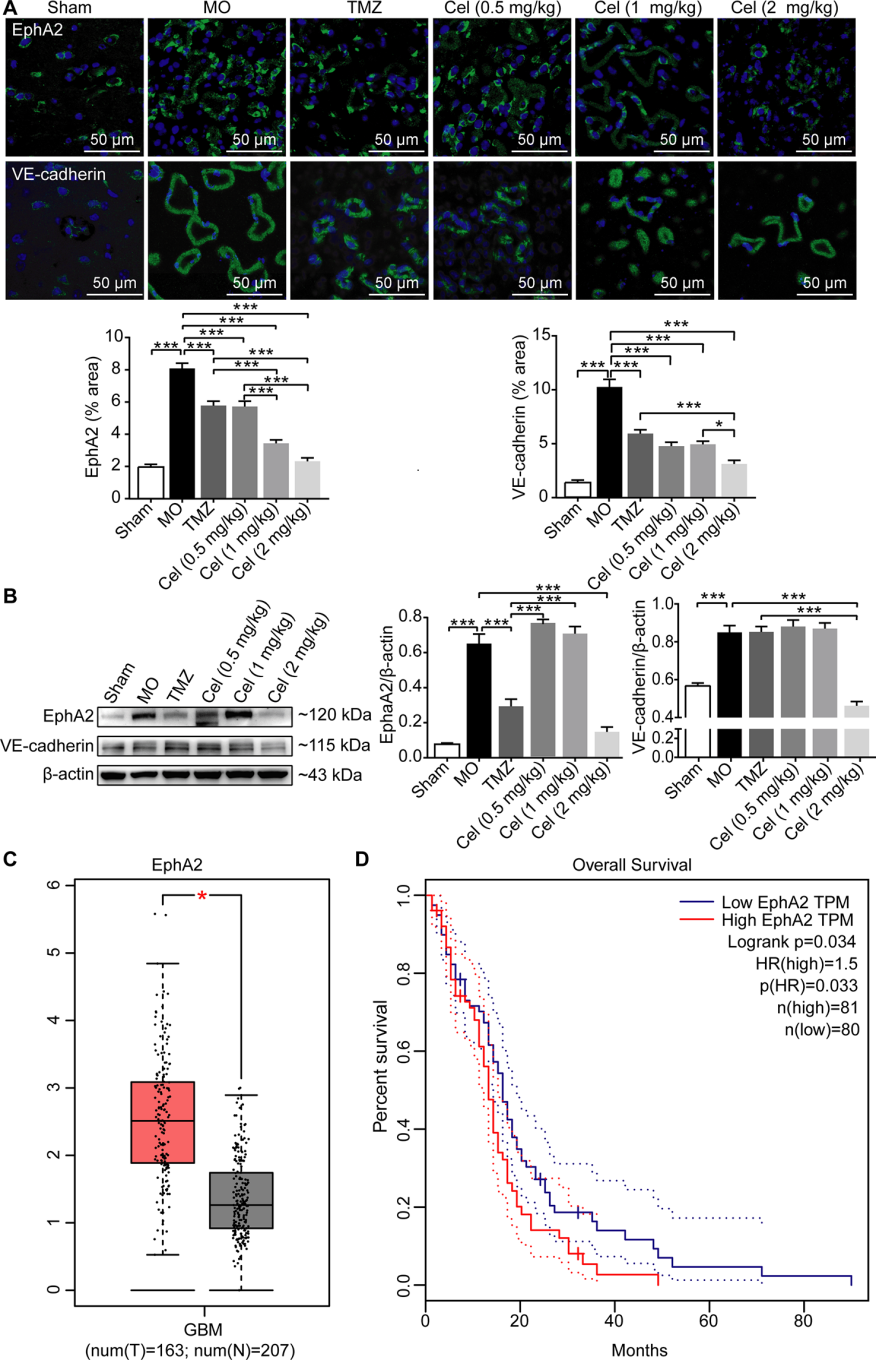
Celastrol decreased the expression of the VM-related proteins EphA2 and VE-cadherin in glioma orthotopic xenografts. **(A)** Immunofluorescence localization of EphA2 and VE-cadherin in tumor vessels. EphA2 and VE-cadherin (green) were labelled with fluorescent secondary antibodies, and the nuclei were labelled with DAPI. **(B)** Western blot analysis of EphA2, VE-cadherin expressions in tumor tissues. **(C)** The expression of EphA2 in tumor patients and normal adults. GBM (glioblastoma), T (tumor patients), N (normal adults). **(D)** The survival analysis of EphA2 via GEAPIA Database. The data are expressed as the mean ± SEM of three independent experiments, *P < 0.05, ***P < 0.001.

Several genes are involved in VM formation, including EphA2 and VE-cadherin (Wykosky et al., 2005; Mao et al., 2013). To further investigate the effect of celastrol on VM, the expression levels of EphA2 and VE-cadherin in tumor tissues were determined by IF and WB analysis. EphA2 and VE-cadherin levels were significantly higher in MO mice than in Sham mice (**Figures 4A, B**). Treatment with celastrol effectively downregulated the expression of EphA2 and VE-cadherin, especially at dose of 2 mg/kg. Then, we searched the GEPIA database and found bioinformatics information indicating that EphA2 expression was significantly higher in GBM tumor tissues than in normal tissues (**Figure 4C**). Additionally, survival analysis revealed that patients with high EphA2 levels had lower survival rates than those with low EphA2 levels, indicating that EphA2 may promote glioma progression (**Figure 4D**). Therefore, the anti-tumor effects of celastrol may be attributable to inhibition of VM formation.

### Celastrol Downregulated the Phosphorylation of Members of the PI3K/AKT/mTOR Signaling Pathway in Glioma Orthotopic Xenografts

**Figure 5:**
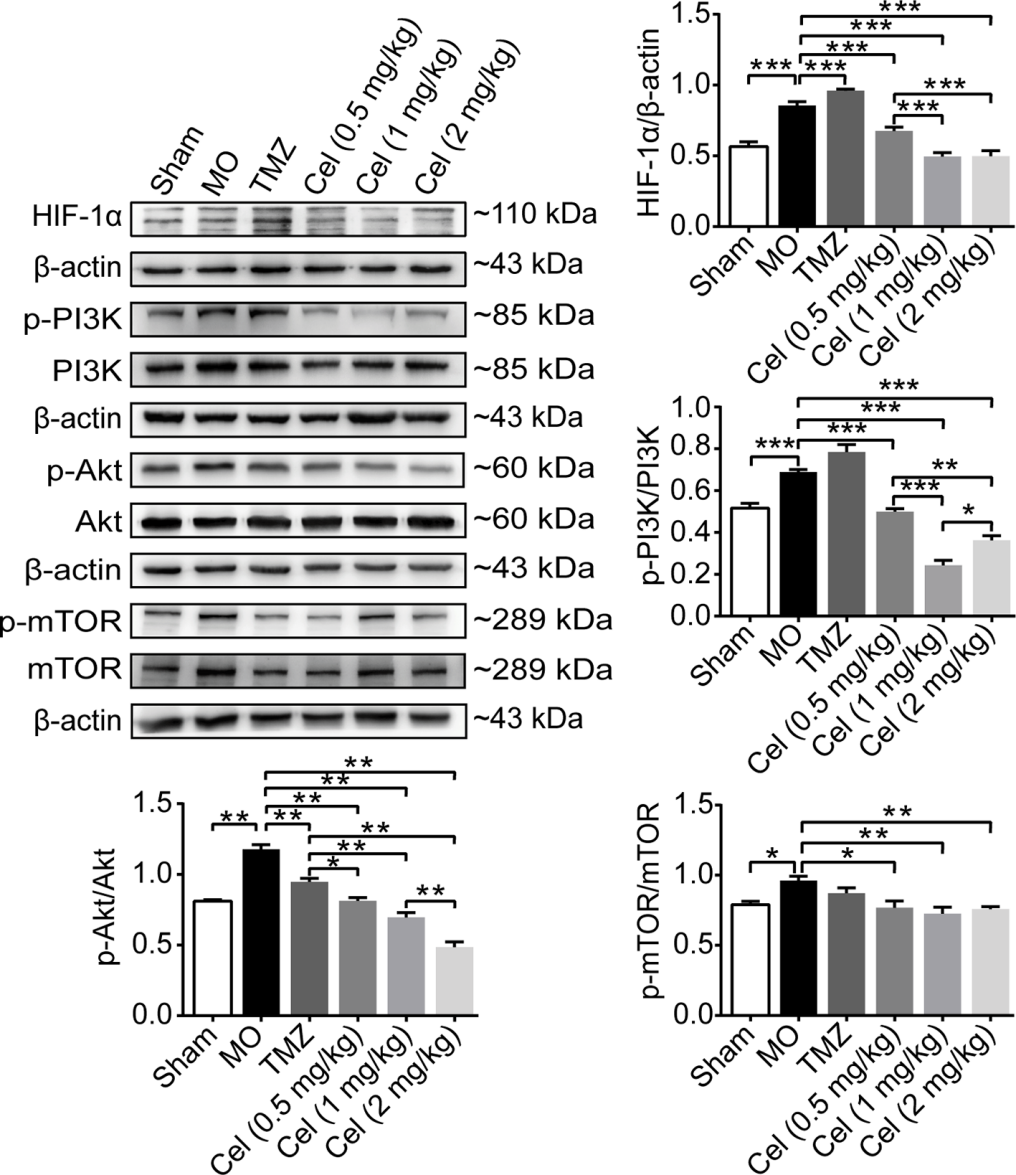
Celastrol downregulated the phosphorylation of members of the PI3K/Akt/mTOR signalling pathway in glioma orthotopic xenografts. Western blot analysis showed the HIF-1α, p-PI3K, p-Akt and p-mTOR expressions in tumor tissues. The data are expressed as the mean ± SEM of three independent experiments, *P < 0.05, **P < 0.01, ***P < 0.001.

Hypoxia is the most frequently observed phenomenon in tumors because of the need for tremendous amounts of energy and oxygen. HIF-1α is a nuclear factor expressed in tumors in response to hypoxia and can directly modulate the expression of VE-cadherin, VEGFR2, and VEGFA (Seftor et al., 2012). PI3K/Akt/mTOR signaling has been regarded as a key in regulator of VM and angiogenesis. In the present study, the expression levels of HIF-1α, phospho-PI3K, phospho-Akt, and phospho-mTOR were all substantially higher in MO mice than in Sham mice (**Figure 5**). In addition, the expression of phospho-Akt was significantly lower in TMZ mice than in MO mice. Treatment with celastrol significantly reduced HIF-1α, phospho-PI3K, phospho-Akt, and phospho-mTOR levels in mice. Our findings suggest that celastrol acts at least in part *via* this cellular pathway to suppress glioma progression.

### Celastrol Reduced the Viability and Inhibited the Migration and Invasion of U87 and U251 Cells

**Figure 6:**
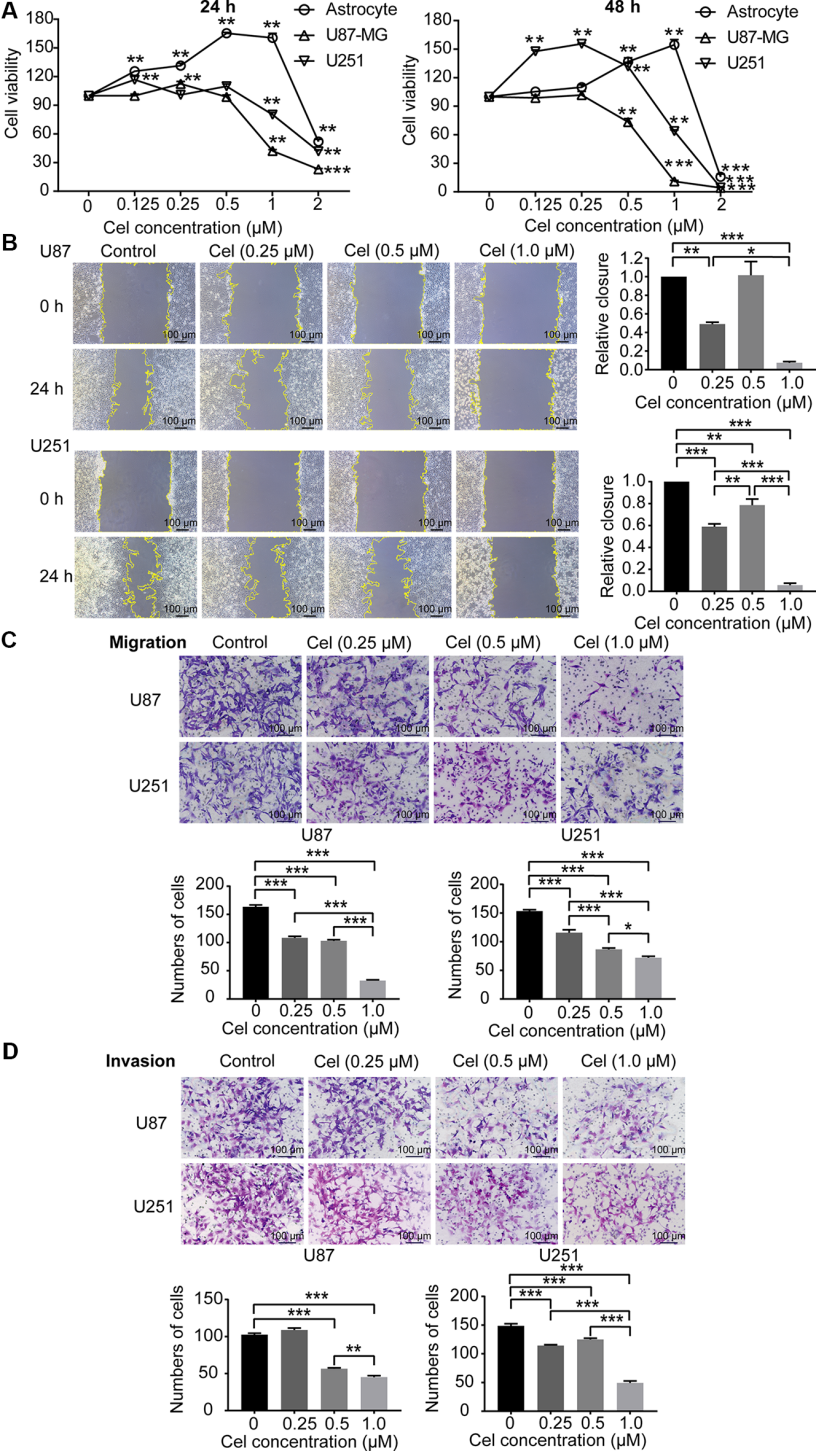
Celastrol (Cel) inhibited the viability and inhibited the migration and invasion of U87 and U251 cells. **(A)** U87 and U251 cells and astrocytes were treated with Cel (0, 0.125, 0.25, 0.5, 1, and 2 μM) for 24 and 48 h. Cell viability was analysed by CCK-8 assay. **P < 0.01, ***P < 0.001, significantly different than the untreated (Cel, 0 μM) group. **(B, C)** Cel inhibited U87 and U251 cell migration according to wound healing and Transwell migration assays. **(D)** Cel impaired U87 and U251 cell invasion, as measured by Transwell invasion assay. The data are expressed as the mean ± SEM of three independent experiments, *P < 0.05, **P < 0.01, ***P < 0.001.

To investigate the cytotoxicity of celastrol on astrocytes, U87 and U251 cells, these cells were treated with celastrol (0.125, 0.25, 0.5, and 1 μM) for 24 and 48 h, and cell viability was measured by CCK-8 assay. Celastrol promoted astrocyte proliferation at concentrations of 0.125–1 μM at 24 and 48 h (**Figure 6A**), while 2 μM celastrol significantly inhibited astrocyte proliferation. The IC_50_ values of celastrol in astrocytes were 1.91 and 2.09 μM for 24 and 48 h of exposure, respectively. Celastrol promoted U87 and U251 cell proliferation at low concentrations of 0.125 and 0.25 μM, the IC_50_ values were 0.80 and 0.60 μM for 24 and 48 h, respectively, in U87 cells, and 1.02 and 0.94 μM for 24 and 48 h, respectively, in U251 cells. According to the data above, celastrol concentrations of 0–1 μM were safe for astrocytes, so we chose 0.25, 0.5, and 1 μM celastrol for further experiments *in vitro*. Migration and invasion are vital for VM formation by glioma cells to form vasculogenic mimicry (Francescone et al., 2011). Wound healing, Transwell migration, and invasion assays demonstrated that celastrol reduced the migration and invasion of U87 and U251 cells. The relative closure in the wound healing assays and the number of migrating and invading cells were quantified (**Figures 6B–D**).

### Celastrol Impaired VM Formation in U87 and U251 Cells

**Figure 7:**
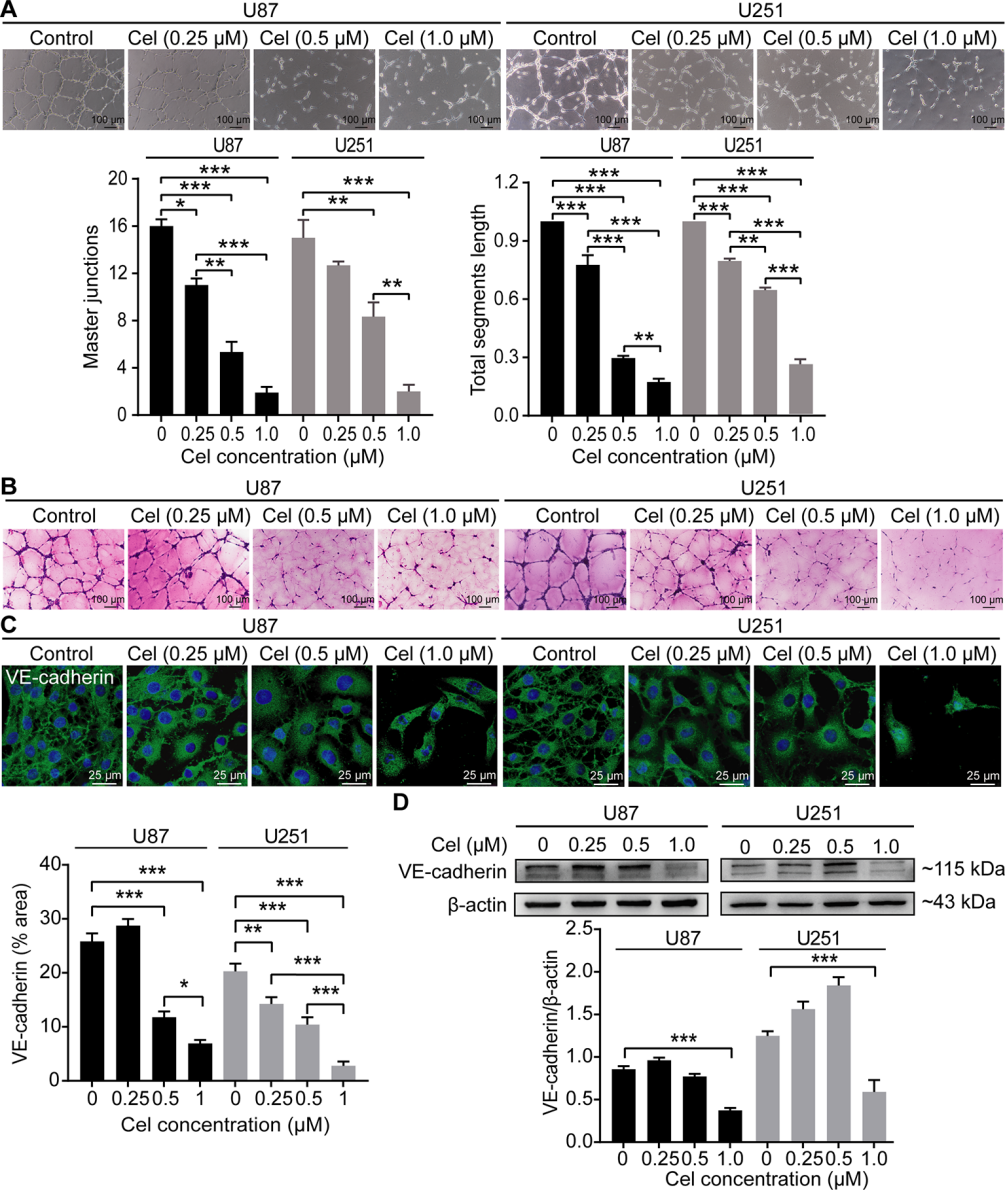
Celastrol (Cel) impaired VM formation in U87 and U251 cells. **(A)** Different concentrations of Cel reduced tube formation by U87 and U251 cells on Matrigel. The average number of master junctions and total segment lengths in four different fields were analysed. **(B)** Periodic Acid-Schiff stain images of U87 and U251 cell cultures on Matrigel. **(C)** Immunofluorescence revealed expression of VE-cadherin to be noticeably downregulated after Cel-treated. VE-cadherin (green) was labelled with fluorescent secondary antibodies, and the nuclei were labelled with DAPI. **(D)** Western blot showed that VE-cadherin levels in cell protein extracts substantially decreased following Cel-treated. The data are expressed as the mean ± SEM of three independent experiments, *P < 0.05, **P < 0.01, ***P < 0.001.

U87 and U251 cells efficiently formed vasculogenic-like networks on Matrigel within 24 h, and celastrol suppressed the formation of these tubular structures in a dose-dependent manner (**Figure 7A**). This effect was confirmed by quantification of master junctions and relative total segments length. Positive PAS staining was observed along the tubules, indicating that the extracellular matrix was involved in VM formation (**Figure 7B**). VE-cadherin is a well-accepted marker of tumor cell-lined VM (Qiao et al., 2015), so the expression of VE-cadherin was monitored in our experiment. Notably, celastrol markedly weakened the expression of VE-cadherin in glioma cell cytoplasm, as observed by IF (**Figure 7C**). In addition, the WB results showed that VE-cadherin was downregulated by treatment with celastrol (**Figure 7D**).

### Celastrol Blocked the PI3K/Akt/mTOR Signaling Pathway in U87 and U251 Cells

**Figure 8:**
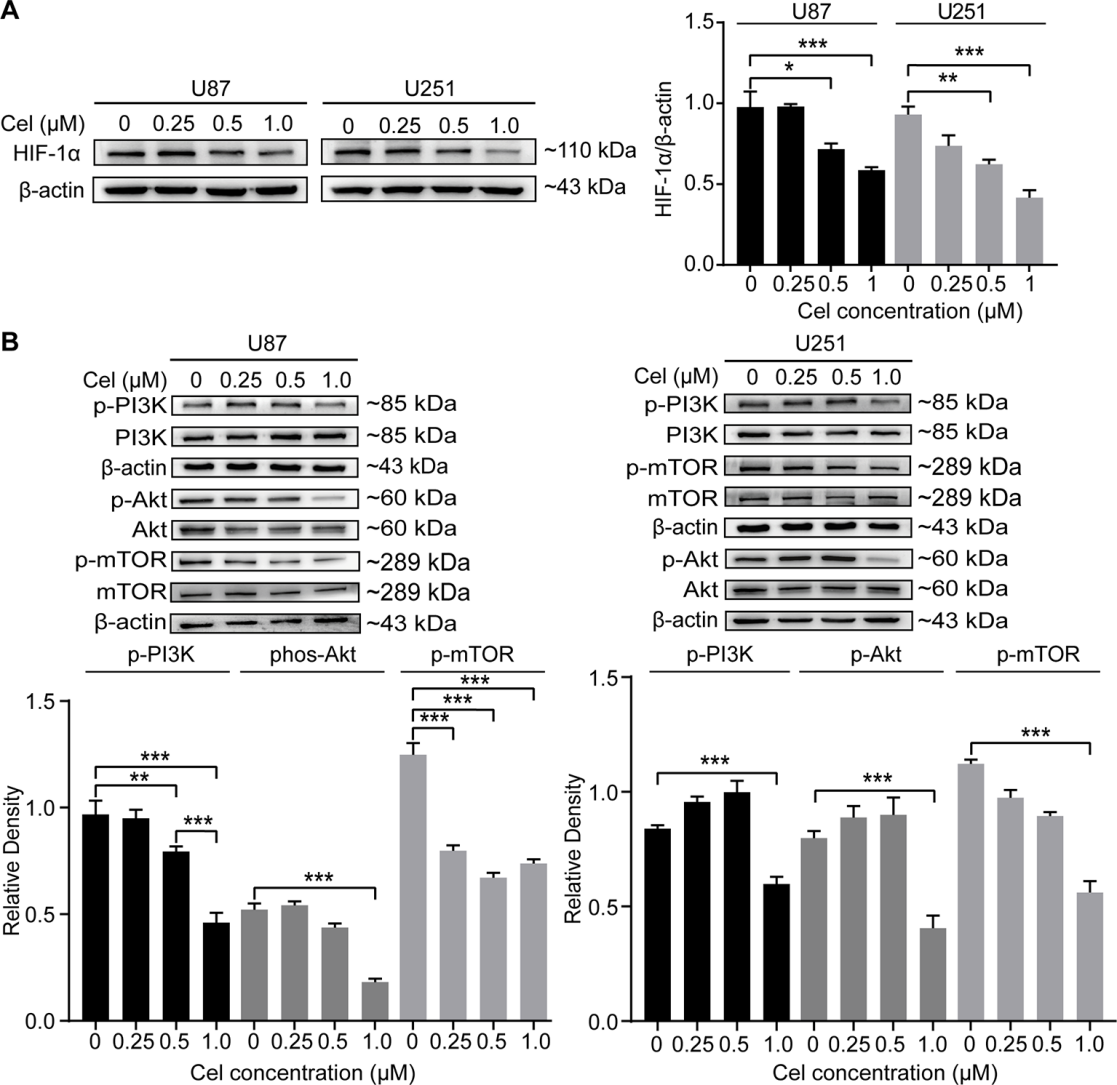
Celastrol (Cel) blocked the PI3K/Akt/mTOR signalling pathway in U87 and U251 cells. **(A, B)** Western blot showed that Cel suppressed HIF-1α levels and the phosphorylation of PI3K, Akt and mTOR in U87 and U251 cells. The data are expressed as the mean ± SEM of three independent experiments, *P < 0.05, **P < 0.01, 71 ***P < 0.001.

HIF-1α can directly regulate VM formation and is associated with poor progression in different tumors (Comito et al., 2011; Sun et al., 2012; Huang et al., 2014; Tang et al., 2014). Our results revealed that 0.5 and 1 μM celastrol obviously suppressed HIF-1α expression in U87 and U251 cells (**Figure 8A**). The effects of celastrol on the PI3K/Akt/mTOR pathway were also detected, and celastrol significantly reduced phospho-PI3K, phospho-Akt, and phospho-mTOR levels in U87 and U251 cells (**Figure 8B**), especially at a concentration of 1 μM.

### Celastrol Interrupted VM Formation in Glioma Cells *via *the PI3K/Akt/mTOR Pathway

**Figure 9:**
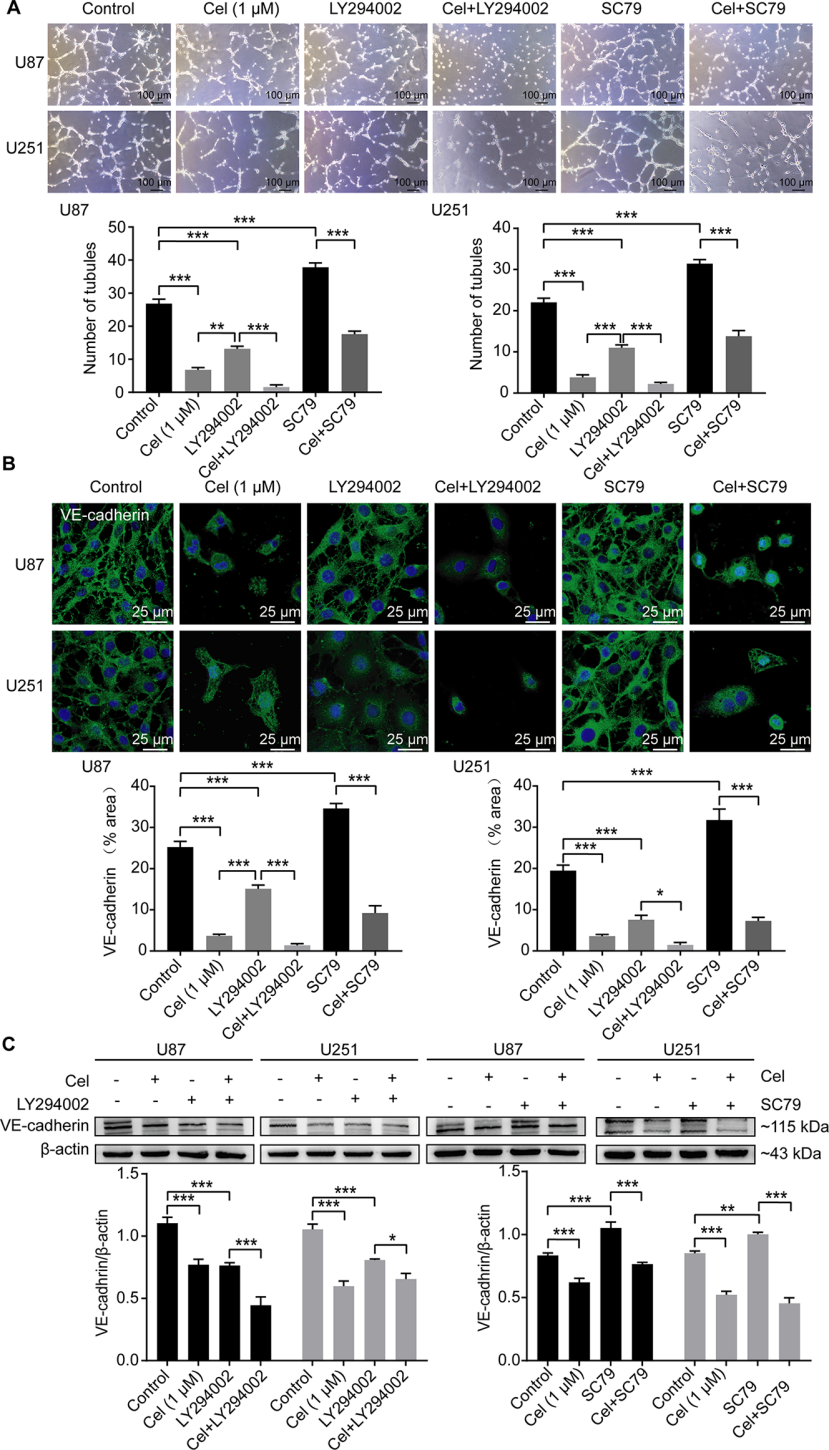
Celastrol interrupted VM formation and VE-cadherin expression in glioma cells via PI3K/Akt/mTOR pathway. **(A)** U87 and U251 cells were treated with 1 μM celastrol (Cel) in the absence or presence of SC79 (8 μg/ml), pretreated with LY294002 (50 μM, 1 h) before treatment of Cel for 24 h. Treatment with Cel and LY294002 decreased number of tubules while SC79 promoted tube formation by U87 and U251 cells on Matrigel. **(B)** Immunofluorescence revealed expression of VE-cadherin to be noticeably downregulated after Cel- and LY294002-treated while upregulated after SC79-treated. VE-cadherin (green) was labelled with fluorescent secondary antibodies, and the nuclei were labelled with DAPI. **(C)** Western blot showed that VE-cadherin was substantially decreased via Cel- and LY294002-treated, while increased via SC79-treated. Proteins in **Figure 10** were used the same β-actin as **Figure 9C** since these proteins were seperated in the same gel. The data are expressed as the mean ± SEM of three independent experiments, *P < 0.05, **P < 0.01, ***P < 0.001.

To investigate whether the PI3K/Akt/mTOR pathway can influence VM formation, U87 and U251 cells were seeded on Matrigel and treated with 1 μM celastrol in the absence or presence of the PI3K inhibitor LY294002 and the novel Akt activator SC79 for 24 h. Treatment with 50 μM LY294002 compromised the ability of glioma cells to form vessels, while treatment with 8 μg/ml SC79 markedly enhanced this ability, as measured by tubules counting (**Figure 9A**). IF and WB analysis confirmed that VE-cadherin was down-regulated in the presence of LY294002, and up-regulated in the presence of SC79 in cell cytoplasm and at the protein level (**Figures 9B, C**).

**Figure 10:**
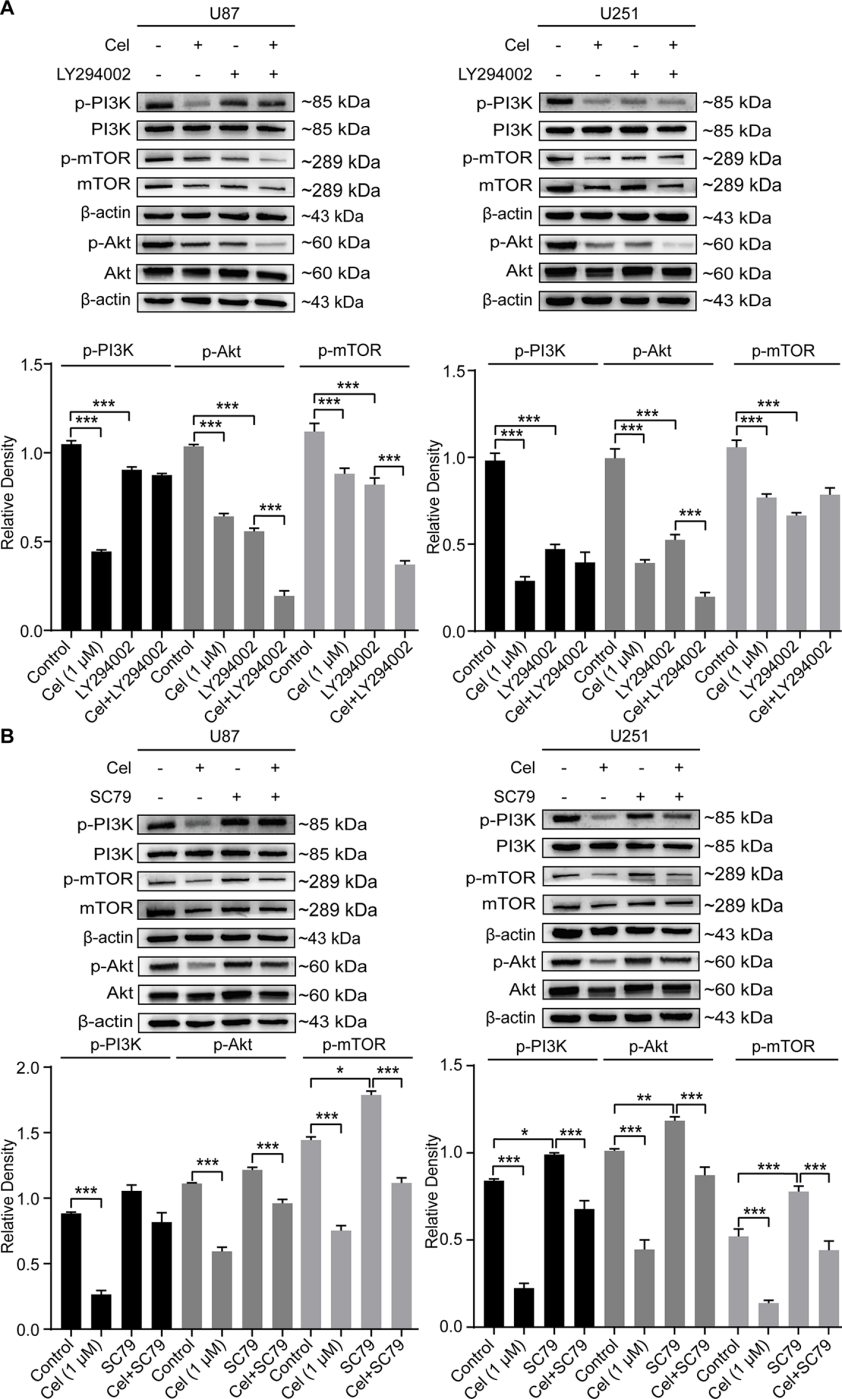
Changes of the phosphorylation of PI3K, Akt and mTOR in glioma cells treated with celastrol (Cel) in the presence of LY294002 or SC79. Western blot analysis showed that p-PI3K, p-Akt and p-mTOR apparently reduced by **(A)** Cel-, LY294002-treated while elevated by **(B)** SC79-treated in U87 and U251 cells. The data are expressed as the mean ± SEM of three independent experiments, *P < 0.05, **P < 0.01, ***P < 0.001.

Treatment with LY294002 effectively reduced the protein levels of phosphorylated PI3K, Akt, and mTOR in U87 and U251 cells, while SC79 increased them (**Figures 10A, B**). Phospho-PI3K, phospho-Akt, and phospho-mTOR levels were significantly lower in cells treated with celastrol in the presence of SC79 than in cells treated with SC79. Taken together, our findings indicate that the inhibition of VM formation by celastrol is in part due to suppression of PI3K/Akt/mTOR signaling in glioma cells.

## Discussion

GBM is one of the most vascularized types of tumors, and an effective blood supply is needed to sustain tumor growth and metastasis (Plate et al., 2012). Unlike endothelium-lined blood vessels, VM channels are formed by tumor cells (Chen et al., 2018). VM channels exist in many types of cancers, including melanoma, hepatocellular carcinoma, small cell lung cancer, sarcoma, and GBM, and are associated with tumor invasion, metastasis, and poor prognosis in cancer patients (Sun et al., 2011; Mao et al., 2015; Williamson et al., 2016; Puerto-Camacho et al., 2019). CVM-1118, a novel molecular compound targeting VM, is currently in clinical trials (Hendrix et al., 2016). VM may represent the main reason for the failure of anti-angiogenic therapy in glioma patients (Wang et al., 2013). Targeting VM formation combined with angiogenesis may effectively block the blood supply to tumors. In this study, our results demonstrated, for the first time, that celastrol might inhibit VM formation and angiogenesis in glioma orthotopic xenografts and glioma cells by regulating the PI3K/Akt/mTOR signaling pathway.

Our previous *in vivo* experiment revealed that 2 and 4 mg/kg celastrol inhibited tumor growth and did not cause major organ-related toxicity in the U251 orthotopic xenograft nude mouse model (Liu et al., 2019). In the present study, 1 and 2 mg/kg celastrol decreased the infiltration and tumor volumes of U87 orthotopic xenografts in nude mice, suggesting that celastrol inhibits glioma growth in animal models, which is consistent with the results of the previous study.

Subsequently, we analyzed the effects of celastrol on microvascular structures in the brain. The delivery of chemotherapy drugs for glioma treatment is restricted by the BBB, and ZO-1, a tight junction-related protein, is the main molecular component of the BBB (Bicker et al., 2014). Furthermore, caveolae are plasma membrane invaginations involved in vesicle endocytosis, and caveolin-1 is an isoform related to BBB permeability regulation (Andreone et al., 2017). Our study proved that celastrol disrupted the basal lamina of the microvasculature and suppressed the expression of ZO-1 in tumor tissues. Membrane protrusions and caveolin-1 expression were also increased in celastrol-treated mice. These results indicate that celastrol probably disrupts vascular structures and promotes the caveolae-mediated transcellular pathway to pass through the BBB.

Most importantly, inhibitory effects of celastrol on VM formation and angiogenesis were identified in glioma orthotopic xenografts. Previous studies have reported that VE-cadherin plays a vital role in the reversion of plastic tumor cells to an embryonic-like state and the adaption of endothelial characteristics, and VM tubules cannot be formed without VE-cadherin (Delgado-Bellido et al., 2019). The EphA2 gene is one of the ephrin subfamily of receptor tyrosine kinases (RTKs) and is involved in promoting cell proliferation, migration, and angiogenesis (Hendrix et al., 2003). It has been reported that activation of EphA2 with the ligand ephrin-A1 inhibits integrin function and induces focal adhesion kinase (FAK) inactivation, resulting in suppression of cell spreading and migration in PC-3, NIH 3T3, and Cos-7 cells (Miao et al., 2000). Additionally, integrin β1-mediated FAK signaling is positively correlated with VM formation in HT1080 cells (Kawahara et al., 2018). Moreover, FAK has been reported to regulate VE-cadherin Tyr658 phosphorylation (Jean et al., 2014). Unlike ligand-mediated activation of EphA2, Akt-mediated phosphorylation of EphA2 on Ser897 has been reported to enhance cell migration and invasion in glioma cells. Both activated Akt and Ser897-phosphorylated EphA2 are robustly expressed in GBM tissues, and an interaction of EphA2 with AKT is associated with the malignant progression of glioma (Miao et al., 2009). Instantaneous knockout of EphA2 in aggressive melanoma cells abrogates tubular structure formation in these tumor cells (Hendrix et al., 2003). Recently, EphA2 expression has been reported to be significantly correlated with VM and to contribute to poor prognosis in breast cancer patients (Mitra et al., 2019). VE-cadherin and EphA2 are both over-expressed in glioma, playing vital roles in VM network formation by promoting extracellular matrix remodeling (Wykosky et al., 2005; Wu et al., 2011; Mao et al., 2013). VEGF is known as the most crucial angiogenin during angiogenesis in glioma. It can also act as a VM promoter, inducing EphA2 to increase matrix metallopeptidases expression and remodel the extracellular matrix (Huang et al., 2014; Liu et al., 2015; Angara et al., 2017). VEGFA has been reported to regulate the proliferation, migration, and survival of vascular ECs by activating its receptor VEGFR2 on ECs along with Ang2 (Fidler and Ellis, 1994; Smith et al., 2015). A previous study has demonstrated that VEGFR2 played a key role in sustaining the “stemness” of glioma stem-like cells during VM formation and tumor initiation (Scully et al., 2012; Yao et al., 2013). The hypoxic microenvironment contributed to maintaining the stem cell-like phenotype in GBM cells and tumors (Platet et al., 2007; Soeda et al., 2009; Pistollato et al., 2010). In addition, HIF-1α has been reported to be associated with invasion, angiogenesis, and VM in GBM (Brat and Van Meir, 2004; Arbab et al., 2015; Colwell et al., 2017). Our study showed that celastrol obviously reduced the numbers of VM channels and EVs and the expression of CD31, VEGFR2, Ang2, VEGFA, EphA2, and VE-cadherin in glioma xenograft tissues, indicating that celastrol might suppress tumor growth by inhibiting angiogenesis and VM formation in glioma. Furthermore, we demonstrated that celastrol reduced HIF-1α expression, and the phosphorylation of PI3K, Akt, and mTOR in U87 orthotopic glioma xenografts.

It is well known that multiple cellular-activated processes are involved in tube formation *in vitro*, and loss of cell-cell adhesion and cell migration are essential during VM (Francescone et al., 2011). Our study proved that, in addition to inhibiting cell migration and invasion, celastrol severely disrupted VM tubes in Matrigel and effectively downregulated VE-cadherin and HIF-1α expression in U87 and U251 cells expression. Subsequently, the mechanism by which celastrol disrupted VM formation was investigated and the results demonstrated that suppression of the PI3K/Akt/mTOR signaling pathway disrupts VM formation in glioma cells (Choi et al., 2014; Zhang et al., 2015). We speculate that celastrol may inhibit VM formation by modulating the PI3K/AKT/mTOR signaling pathway.

Hyperactivation of the PI3K/Akt/mTOR pathway in GBM affects various biological processes, including cell proliferation, apoptosis, cytoskeletal rearrangement, angiogenesis, and VM formation. Dysregulation of this pathway is also related to the therapeutic resistance of glioma (Khan et al., 2012). The limited effects of pan-PI3K inhibitors and rapamycin analogs in the clinic provide a rationale for trials with drugs targeting both PI3K and mTOR for glioma treatment (Chang et al., 2005; Galanis et al., 2005). The PI3K regulatory subunit p85, which leads to the activation of the catalytic subunit p110, causes phosphatidylinositol 3,4-bisphosphate (PIP2) to be phosphorylated, forming phosphatidylinositol 3,4,5-trisphosphate (PIP3) (Cantley, 2002). Subsequently, PIP3 activates phosphoinositide-dependent kinase (PDK) 1 and Akt by phosphorylation at the Thr308 and Ser473 residues (Vivanco and Sawyers, 2002). Activated Akt, in turn, phosphorylates the downstream molecule mTOR to initiate protein synthesis (Sami and Karsy, 2013). The present study demonstrated that celastrol reduced phosphorylated PI3K, Akt, and mTOR levels in U87 and U251 cells. Furthermore, SC79, an Akt activator, promoted VM formation and the expression of VE-cadherin, indicating that the Akt pathway facilitated these processes. Additionally, treatment with celastrol in the presence of SC79 or LY294002, a PI3K inhibitor, impaired VM in glioma cells. In conclusion, these observations suggest that celastrol likely interrupts VM formation in U87 and U251 cells partly by blocking the PI3K/AKT/mTOR pathway. Taken together, the data indicate that EphA2 and VE-cadherin may promote VM formation in glioma through FAK phosphorylation and PI3K/Akt activation. We will further determine whether celastrol causes FAK dephosphorylation to prevent the VM formation in future work.

**Figure 11:**
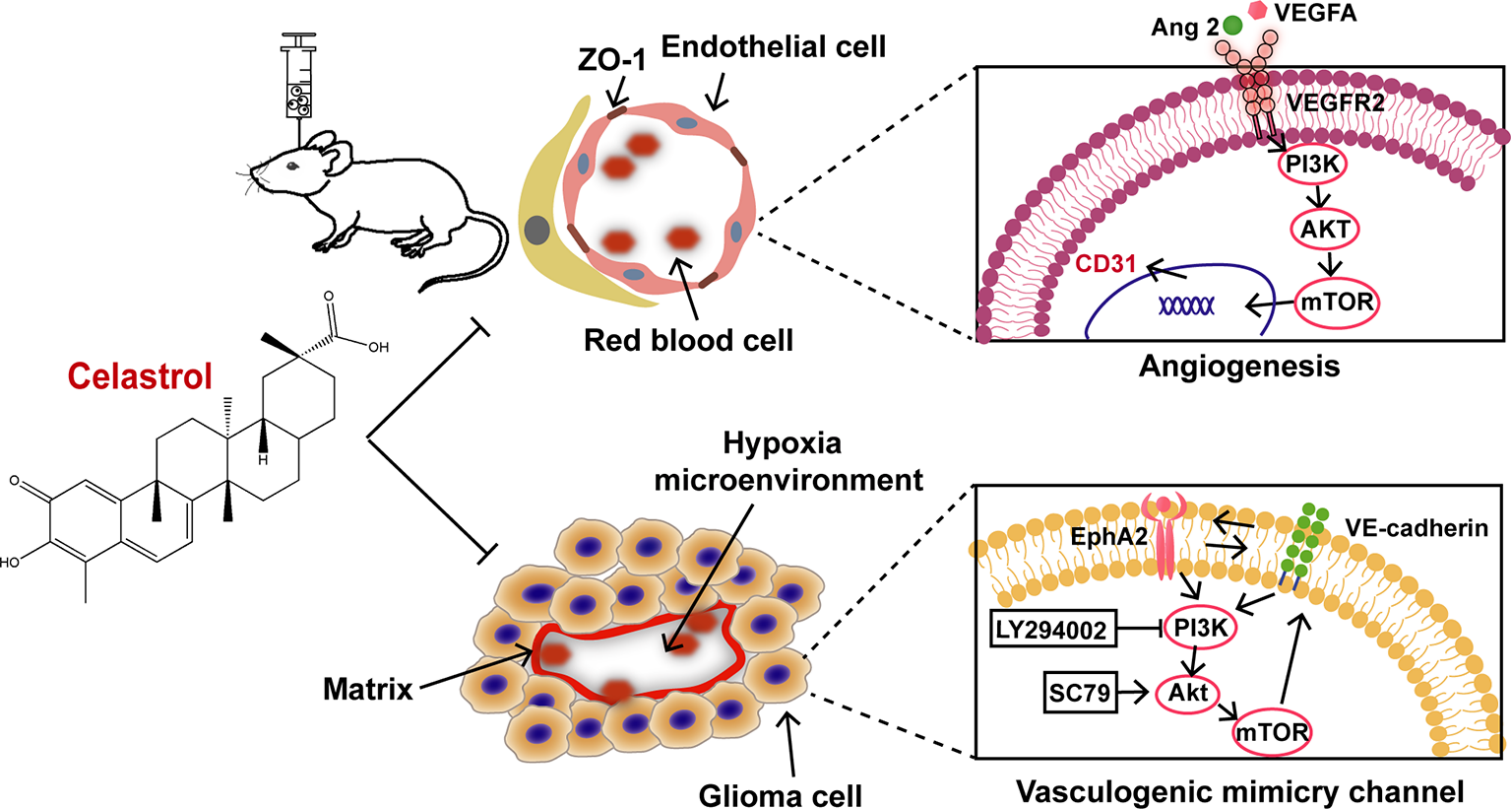
Proposed mechanism of celastrol-suppressed VM and angiogenesis in glioma.

The current study indicates that celastrol inhibits the growth, angiogenesis, and VM formation of glioma through a mechanism likely related to suppression of the PI3K/Akt/mTOR pathway (**Figure 11**). In agreement with previous studies, celastrol induced cell cycle arrest, prompted apoptosis, triggered autophagy, disrupted VM formation and angiogenesis in glioma. All the findings above imply that celastrol elicits synergistic effects in anti-tumor treatment. The exact anti-glioma mechanisms of celastrol remain to be further explored.

## Data Availability Statement

All datasets generated for this study are included in the article/**Supplementary Material**.

## Ethics Statement

The animal study was reviewed and approved by the Animal Experiments and Experimental Animal Welfare Committee of Capital Medical University.

## Author Contributions

YZ and LW designed the study. YZ, XL, and PZ performed the experiments. YZ and LW analyzed the data. YZ wrote the manuscript. HZ and WG revised the manuscript. All authors approved the final manuscript. LW and WG supported the funding. LW and WG supported the funding.

## Funding

This work was supported by a grant from the Beijing Municipal Education Commission of China (No. 17ZY01), the High-level Teachers in Beijing Municipal Universities in the Period of the 13th Five-year Plan (CIT&TCD20140329, CIT&TCD20170324), the National Program for Special Support of Eminent Professionals, the National Natural Science Foundation of China (81973418), and the Scientific Research Key Program of Beijing Municipal Commission of Education (KZ201710025022).

## Conflict of Interest

The authors declare that the research was conducted in the absence of any commercial or financial relationships that could be construed as a potential conflict of interest.

## Supplementary Material

The Supplementary Material for this article can be found online at: https://www.frontiersin.org/articles/10.3389/fphar.2020.00025/full#supplementary-material
