# Supplementary material for: Celastrol Suppresses Glioma Vasculogenic Mimicry Formation and Angiogenesis by Blocking the PI3K/Akt/mTOR Signaling Pathway
Source: Front Pharmacol. 2020 Feb 6;11:25. doi: 10.3389/fphar.2020.00025 (PMC7025498; doi:10.3389/fphar.2020.00025)
Supplement: Supplementary file 1 [file DataSheet_1.pdf]

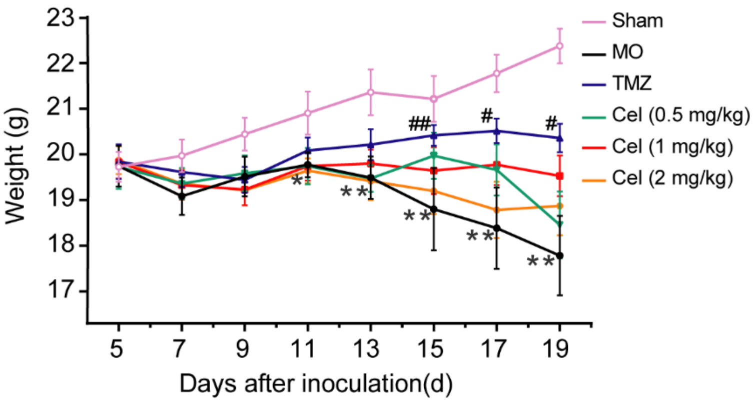

Supplementary Figure 1. Body weigh change in mice during the time of intraperitoneal injection of corresponding drugs. Data expressed as mean ± SEM, $^{*}P< 0.05$, $^{**}P<0.01$ *vs* sham operated (Sham) group. $^{\#}P<0.05$, $^{\#\#}P<0.01$ *vs* model (MO) group.

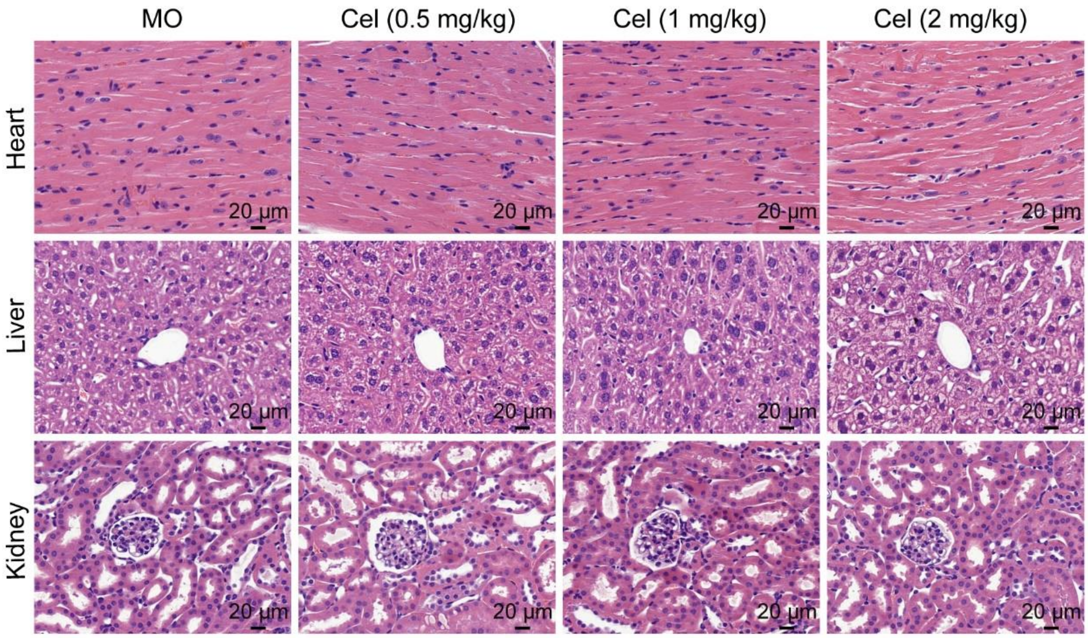

Supplementary Figure 2. H&E staining of heart, liver and kidney for analysis of celastrol toxicity.
